# Mismatched unrelated donor allogeneic stem cell transplant for high risk haematological malignancy: A single centre experience

**DOI:** 10.1038/s41408-017-0012-0

**Published:** 2017-12-15

**Authors:** Heshani Mediwake, Cameron Curley, Jason Butler, Angela Mclean, Siok Tey, Geoffrey R. Hill, Anthony Morton, Ashish Misra, Elango Subramoniapillai, Simon Durrant, Glen A. Kennedy

**Affiliations:** 1 Department of Bone Marrow Transplantation, Royal Brisbane and Women’s Hospital, Brisbane, 4029 Australia; 2 School of Medicine, University of Queensland, St Lucia, Queensland Australia; 3 QIMR Berghofer Medical Research Institute, Brisbane, Queensland 4006 Australia

Human leukocyte antigen (HLA) matched allogeneic stem cell transplant (SCT) is an established curative treatment for various haematologic malignancies^1^. However, a fully matched HLA-donor may still not be identified in a significant proportion of patients. Those who do not have an available HLA-matched donor can still potentially undertake transplantation utilizing an alternative donor source, including mismatched unrelated donors (MMUD), umbilical cord blood (UCB) or haploidentical donors^2^. Although MMUD SCT is associated with increased risk of both graft versus host disease (GVHD) and non-relapse mortality (NRM), MMUD have still historically been utilized as alternative donor sources due to ease of donor availability^3^.

Historically, our Unit has offered MMUD SCT to individuals with high-risk haematological malignancies without an available HLA-matched donor. We aimed to review the outcome of consecutive patients undertaking MMUD SCT for acute leukaemia and myelodysplastic syndrome (MDS)/myeloproliferative neoplasm (MPN) performed at our institution between January 2004 and December 2014. Consent for release of information was obtained as per approved institutional practice for clinical audit. Conditioning protocols included myeloablative (cyclophosphamide 120 mg/kg and 12 Gy TBI or melphalan 140 mg/m^2^ and 12 Gy TBI) and reduced intensity conditioning (fludarabine 125 mg/m^2^ plus melphalan 120 mg/m^2^). All grafts were T-replete, with no use of ATG/Alemtuzumab, and peripheral blood progenitor cells (PBPC) used as stem cell source in all cases. GVHD prophylaxis consisted of cyclosporine and day 1, 3 6 and 11 methotrexate ± administration of a single dose of Tocilizumab (TCZ) of 8 mg/kg at day-1 of SCT as part of a prospective phase II study being conducted at the time. Prior to 2008, class I typing was performed using low resolution sequence specific primers (SSP), and high resolution sequence based typing (SBT) methods used only for HLA class II alleles. After 2008, SBT methods were utilized for both class I and II typing. Survival analysis was calculated from day of transplant to relapse or last follow up for progression free rate (PFR), and to death or last follow up for OS. NRM, acute GVHD incidence and GVHD-free, relapse-free survival (GRFS)^4^ were determined from the day of transplant. Survival analysis was performed using the Kaplain-Meier method. Acute and chronic GVHD were classified according to the Seattle criteria^5, 6^.

In total 52 patients underwent MMUD SCT for AML/MDS/MPN during the time period under review, representing 7.7% of all allogeneic SCT performed at our institution for these indications over this time. Baseline characteristics of are shown in Table 1. In total, 40 donor/recipient pairs (77%) were mismatched at 1 loci only, including 23 with mismatches at HLA class I (44%) and 17 (33%) at class II loci. Mismatches at > 1 loci occurred in 12 cases (23%), including 7 cases involving C-mismatches (C + B, *n* = 2; C + DRβ1, *n* = 2; C + A, *n* = 1; C + DQ, *n* = 1; C + B + DQ, *n* = 1), and 5 cases involving DQ mismatches (DQ + DRβ1, *n* = 3; DQ + A, *n* = 1; DQ + B, *n* = 1). Of the 12 multi-mismatched donor/recipient pairs, class 1 HLA-mismatches were identified by low resolution typing in 8 of 9 cases with class 1 mismatches present. For the whole cohort, direction of mismatch was bidirectional in 49 cases (94%) and in a “host versus graft” direction in three cases (6%), including × 2 single mismatched donor/recipient pairs (single C + DQ mismatches respectively) and × 1 multi-mismatched donor/recipient pair (B + C mismatched pair).

**Table 1:** Patient demographics and SCT details

Total	*N* = 52
Age (median; range)	45 yrs (range 17–65yrs)
Gender	
Male	23 (44%)
Female	29 (56%)
Disease	
AML	31 (59%)
ALL	16 (31%)
MDS/MPN	5 (10%)
Risk category^a^	
AML	
Poor risk cytogenetics (including > CR1/2nd AML)	9 (5)
FLT3 ITD positive (including > CR1)	4 (1)
>CR1 without poor risk cytogenetics (including 2nd AML)	8 (3)
2nd AML in CR1 (without poor risk cytogenetics)	10
ALL	
Poor risk cytogenetics (including high WCC/age >35)	11 (9)
>CR1	5
MDS/MPN	
IPSS intermediate 2 or above	5
Conditioning regimen	
Cy/TBI	32 (62%)
Mel/TBI	1 (1%)
Flu/Mel	19 (37%)
GVHD prophylaxis	
MTX/CSA	45 (87%)
MTX/CSA/TCZ	7 (13%)

*Cy/TBI*   cyclophosphamide/TBI, *Mel/TBI*  melphalan/TBI, *Flu/Mel*   fludaraine + melphalan

*Poor risk cytogenetics defined as per SWOG risk stratification for AML;^11^ *t*(9;22), *t*(4:11), complex karyotype, low hypodiploidy/near triploidy, age >35 yrs, WCC > 35×10^9^/l for B-ALL and >100×10^9^/l for T-ALL for high risk ALL^12–14^, as per IPSS for MDS/MPN^15^

Neutrophil and platelet engraftment occurred in 98% and 94% of patients, respectively, at a median of 16 days (range 11–141 days) and 19 days (range 7–141 days) post-SCT; 3 patients (6%) died prior to platelet engraftment. All but one patient surviving past D28 post-SCT achieved full donor chimerism on short tandem repeats (STR) analysis of nucleated cells in peripheral blood. Overall incidence of grade II–IV and III–IV acute GVHD was 50% and 17%, respectively, with 84% of patients surviving post D100 (*n* = 37) developing chronic GVHD. Chronic GVHD was limited in 9 patients (20% of patients surviving post D100) and extensive in 28 (64% of surviving patients). Of patients with extensive stage chronic GVHD > 2 organs were involved in 89%, including liver in 10 cases (36%) and lungs in 6 (21%).

**Fig. 1:**
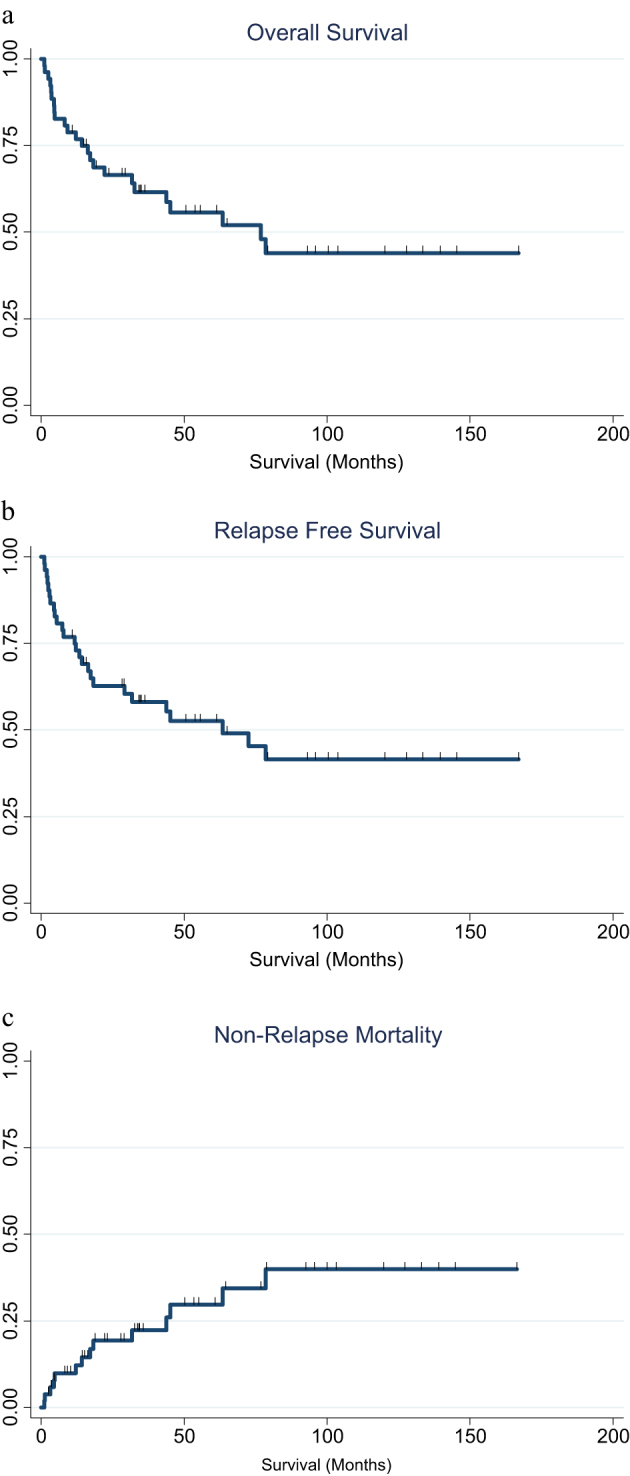
Survival outcomes for patient cohort **a** OS, **b** PFR and **c** NRM.

At median follow-up of survivors of 63mths (range 11–169mths) 5 yr OS, PFR and NRM and was 56%, 53% and 30%, respectively (Fig. 1), with 12 month GRFS 29%. To date 24 deaths have occurred, including 10 related to relapsed disease (42%) and 14 (58%) related to NRM. In total, 13 patients suffered relapsed disease at a median of 8mths post-SCT (range 2–74mths). Causes of NRM included infection (*n* = 6), GVHD (*n* = 4), secondary malignancies (*n* = 2), post-transplant microangiopathy (*n* = 1) and failed engraftment (*n* = 1).

On univariate analysis, the only factor predictive of OS was development of grade III–IV GVHD (2 yr OS 71% versus 44% respectively, *p* = 0.009). There was no association between OS and conditioning intensity, age > versus < median, degree of HLA mismatch or underlying disease. Specifically, no significant difference in OS was noted between HLA class 1 versus class 2 mismatched donors, or single versus multiple HLA-mismatched donors. Although there was no significant difference observed with respect to 12 month GRFS and degree of HLA mismatch, a favourable trend was noted with respect to both improved 12mth GRFS with single versus multiple HLA mismatched donors (HR 1.66 (95% CI 0.91–3.04); *P* = 0.098), as well as HLA class 1 versus class 2 mismatched donors (HR 1.45 (95% CI 0.92–2.23); *p* = 0.11). Treatment with TCZ did not appear to have any impact on reducing incidence of acute or chronic GVHD or 12mth GFRS.

Traditionally, MMUD transplants have been associated with increased rates of acute and chronic GVHD with corresponding poor OS related to high risk of NRM^3, 7^. A majority of studies assessing outcomes post MMUD SCT are from large registries and have included patients receiving ATG as T-cell depletion and/or bone marrow grafts (as opposed to peripheral blood) as stem cell source^3, 7, 8^. Our study differs from these in that we exclusively examined outcomes in consecutive patients undertaking T-replete MMUD SCT with PBSC grafts, i.e., a transplant platform associated with increased risk of GVHD. Given these factors, our 5 yr OS of 56% compares well with larger MMUD SCT series reporting 1 yr and 5 yr OS incidence of 47% and 39%, respectively^3, 8, 9^. In our experience, despite high rates of GVHD, NRM was relatively low at 30%, potentially related to improvements in treatment and supportive care in GVHD over the last several years. The relatively low relapse rate realized in our cohort highlights the potential high curative potential of MMUD transplantation in high-risk malignancies, albeit at a cost of high risk of GVHD. Although patient numbers were low, administration of a single dose of TCZ at day -1 of SCT did not appear to have any impact on reducing incidence of acute or chronic GVHD or GFRS.

Previously published data have suggested that mismatch at HLA class 1 alleles is especially associated with significantly reduced OS when compared to matched unrelated donor transplants, and that presence 2 versus 1 HLA-mismatches are also associated with inferior outcomes^1, 3, 8–10^. Overall, clinical impact of low *versus* high resolution HLA mismatches on OS, disease free survival, NRM or incidence of acute GVHD appears insignificant^3^, and impact of individual HLA class 1 and class 2 mismatches on survival and GVHD outcomes remains controversial^3, 9^. Likely related to the relatively low number of patients with specific HLA-mismatches within our cohort, we were unable to demonstrate any significant association between degree of HLA mismatch and GVHD incidence and/or OS.

MMUD remain a potentially important source of stem cells for patients with aggressive haematological conditions who do not have a readily available donor. However, despite the encouraging survival rates seen in our cohort, our experience confirms the high risk of both acute and chronic GVHD with use of MMUD. Transplant approaches that reduce GVHD incidence without increasing relapse rates are clearly required. Post-transplant high dose cyclophosphamide is potentially one such option and should continue to be examined as an alternative approach in this setting.
